# Satellite Glial Cells Control Sensory Neuron Excitability via the Release of Fibulin-2

**DOI:** 10.64898/2026.02.13.705760

**Published:** 2026-02-15

**Authors:** Irshad Ansari, Pan-Yue Deng, Sarah F. Rosen, Michael B. Thomsen, Vitaly A. Klyachko, Valeria Cavalli

**Affiliations:** 1 Department of Neuroscience, Washington University School of Medicine, St Louis, MO 63110, USA; 2 Department of Cell Biology and Physiology, Washington University School of Medicine, St Louis, MO 63110, USA; 3 CS27 Bioinformatics, Springboro, OH, USA; 4 Hope Center for Neurological Disorders, Washington University School of Medicine, St. Louis, MO 63110, USA; 5 Center of Regenerative Medicine, Washington University School of Medicine, St. Louis, MO 63110, USA; 6 Pain Center, Washington University School of Medicine, St. Louis, MO 63110, USA

**Keywords:** Dorsal root ganglia, Satellite glial cells, Fibulin-2, extracellular vesicles, Kv4, sensory neuron, excitability, hypersensitivity, pain

## Abstract

Pain remains a major clinical challenge, with current therapies often limited by low efficacy and adverse effects. While excitability of sensory neurons in the dorsal root ganglia (DRG) is central to pain signaling, accumulating evidence highlights the importance of non-neuronal cells in modulating neuronal activity. Here, we identify satellite glial cells (SGCs) as a source of Fibulin-2, an extracellular matrix glycoprotein with diverse roles in nervous system development and repair. Using SGC primary cultures and mass spectrometry, we demonstrate that Fibulin-2 is secreted by SGCs in part via extracellular vesicles. Electrophysiological functional assays reveal that application of recombinant Fibulin-2 to cultured sensory neurons reduces neuronal excitability by modulating Kv4-mediated currents. *In vivo*, loss of Fibulin-2 leads to reduced Kv4.2 and Kv4.3 expression and heightened mechanical, heat and cold sensitivity in mice. Our findings uncover a novel SGC-sensory neuron signaling mechanism modulating pain sensitivity, suggesting Fibulin-2 as a potential therapeutic target for pain management.

## INTRODUCTION

Sensory neurons residing in the dorsal root ganglia (DRG) receive and process a wide range of external and internal stimuli. These neurons mediate responses to physical and chemical stimuli, enabling the perception and discrimination of sensations such as touch, itch, temperature, proprioception and pain. Among those, pain is an adverse sensory experience that can be caused by a variety of stimuli such as nerve damage, cancer, diabetes, infection, chemotherapy and autoimmune diseases ^1–4^. These conditions are the main contributors to chronic pain. Current clinical treatments for chronic pain primarily target sensory neurons, but have low analgesic efficiency and major side effects ^5,6^, highlighting a need for a better understanding of the cellular and molecular mechanisms underlying pain conditions in order to develop alternative approaches to alleviate chronic pain.

While DRG neurons are the primary transmitters of sensory information and play a key role in the maintenance and processing of pain signals, their activity is also regulated by the local microenvironment. Non-neuronal cells have recently emerged as critical partners in the transmission of sensory information, which requires the integrated function of multiple cell types, including satellite glial cells (SGCs), fibroblasts and macrophages ^2,7,8^. Indeed, in rodent models, numerous cellular, molecular and gene expression changes in these cells have been implicated in various painful states ^7–13^. Recent single-cell multi-omic analyses of human DRG further support the potential implications of multiple non-neuronal cell types in peripheral neuropathies ^14,15^.

Within the DRG, the soma of each sensory neuron is tightly enveloped by multiple SGCs ^16,17^. This unique cellular arrangement forms an environment that modulates neuronal function through potassium buffering and bidirectional communication ^7,9,17^. SGCs have emerged as key players underlying multiple pain conditions ^9,13,18^. Under pathological conditions such as mechanical or chemical nerve injuries, SGCs undergo transcriptional changes that support axon regeneration ^19–21^ or modulate pain development ^9,22^. Many studies have indicated that SGCs contribute to regulating pain thresholds by controlling extracellular potassium levels via their expression of inward rectifier potassium channels Kir4.1 ^10,23–25^. Another way by which SCGs regulate pain thresholds is via ATP signaling, which acts on purinergic receptors in both sensory neurons and SGCs ^7,9^. A recent study unraveled an additional layer of regulation of pain thresholds via the release of the protein diazepam binding inhibitor (DBI) by SGCs ^26^. DBI acts as a positive allosteric modulator of neuronal GABA_A_ receptors, which are expressed by a subpopulation of mechanosensitive sensory neurons ^26^. These finding underscores the pivotal role of SGC-derived factors in the modulation of sensory neuron activity. However, the specific molecules that make up the SGC secretome have yet to be identified, and their potential influence on neuronal excitability and contribution to pain remain largely unexplored.

Dysregulation of the extracellular matrix (ECM) proteins has emerged as a contributor to inflammatory and neuropathic pain in both rodent models and humans ^27,28^. For example, fibroblasts within the DRG contribute to neuropathic and mechanical nociception via secretion of proteins that incorporate into basement membrane enveloping the SGC/neuron sensory units ^29^. SGCs have also been recently suggested to modulate the ECM within the DRG microenvironment ^22,30^. Fibulin-2 is an ECM glycoprotein crucial for the formation and stabilization of elastic fibers, the essential constituents of the ECM of higher vertebrates for tissue elasticity and resilience, and basement membranes ^31,32^. In addition, Fibulin-2 has been shown to play several roles in both the central and peripheral nervous system development ^33,34^. In the dorsal spinal cord, increased Fibulin-2 expression contributes to neuropathic pain by inhibiting the GABA_B_ receptors expressed by sensory neurons terminals ^35^. Fibulin-2 has also been implicated in disease conditions like multiple sclerosis, via its role in inhibiting the maturation of oligodendrocyte progenitor cells ^36^. While emerging evidence points to Fibulin-2 function beyond elastic fibers in the nervous system, its role in the DRG microenvironment and pain modulation has not yet been explored.

In this study, we used proteomic profiling to define the secretome of purified cultured SGCs and identified Fibulin-2 as an SGC-secreted protein. Combining electrophysiological, biochemical and behavioral assays revealed that SGC-derived Fibulin-2 reduces sensory neuron excitability and firing frequency, while Fibulin-2 loss contributes to decreased pain thresholds, highlighting the importance of Fibulin-2 in SGC-neuronal communication in normal and pain conditions.

## RESULTS

### SGCs secrete the ECM protein Fibulin-2

Recent evidence suggests that SGCs can modulate sensory neuron functions via release of small molecules, such as ATP ^7,9,37^ or proteins such as DBI ^26^, but the ensemble of proteins that make up the full SGC secretome have yet to be identified. To identify the repertoire of proteins secreted by SGCs, we cultured primary SGCs following an established protocol ^38^. At 8 days in vitro (DIV8), cultured SGCs were immunostained with established SGC markers, including FABP7 and Glutamine synthetase (GS), as well as TUJ1 (neuronal marker) and PDGFRα-positive cells (fibroblast marker) (Fig. S1A), validating the identity and purity of SGC cultures, as described ^38^. We also performed real-time quantitative PCR (RT-qPCR), further supporting the identity and purity of SGCs cultures (Fig. S1B). Because cultured SGC adopt a spindle-shaped morphology that is distinct from their *in vivo* morphology ^38^, we sought to confirm that cultured SGCs are representative of SGCs *in vivo* by performing a transcriptomic correlation analysis of RNA-sequencing data from primary mouse SGC cultures (obtained at DIV8, 24h after switching or not to serum-free media) with SGC signatures from DRG single cell data ^39^. This analysis revealed that at the transcriptional level, cultured SGCs had the highest similarity to glial cells (SGCs and Schwann cells) but also shared similarities with fibroblasts and mural cells (Fig. S1C), which could reflect the loss of their *in vivo* arrangement surrounding neuron soma. We noted that the 24h serum-free treatment showed slightly elevated similarity to fibroblasts compared to serum-containing cultures. We also analyzed expression of SGC-specific genes in the RNA-sequencing from primary mouse SGC culture, which revealed similar results to RT-qPCR (Fig. S1D). These findings support the validity of using primary SGC cultures to study their properties *ex vivo*.

**Figure 1:**
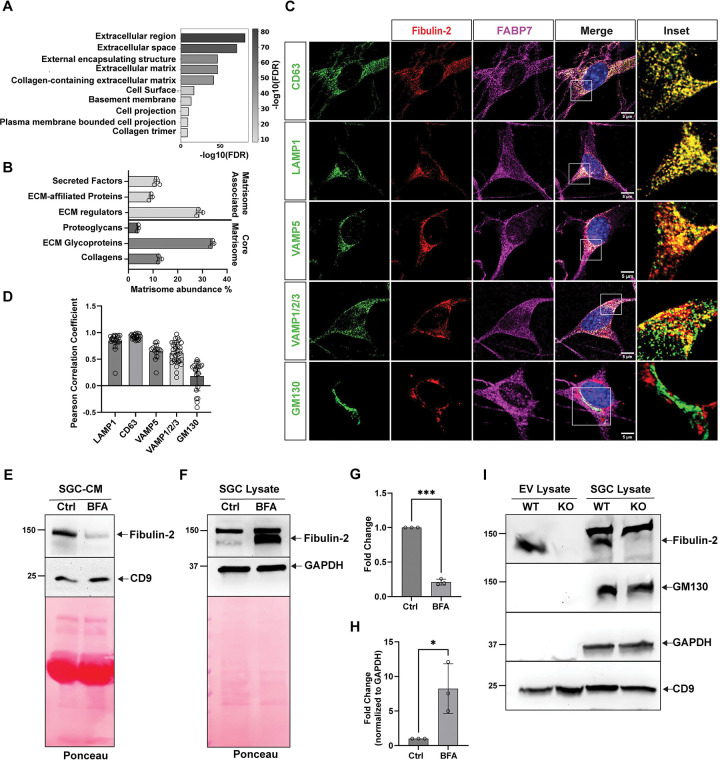
Fibulin-2 is secreted by SGCs **A.** GO pathway analysis (cellular component) of the SGC-secreted proteins showing enrichment for extracellular proteins. **B.** SGC-secreted proteins were analyzed using the matrisome analyzer. **C.** Representative images of cultured SGCs immunostained for Fibulin-2, along with the indicated cellular markers. Lamp1-Lysosome, CD63-multivesicular bodies, VAMP1/2/3 (note that the antibody recgonizes VAMP1, VAMP2 and VAMP3) and VAMP5- secretory vesicles, and GM130- Golgi apparatus. 10–15 fields per group were imaged using confocal microscopy. **D.** Quantification of the Pearson correlation coefficient for the co-localization of Fibulin2 with the different cellular compartment markers. Each data point in the bar graph represents the colocalization coefficient from an individual cell (LAMP1 n=25 cells, CD63 n=25 cells, VAMP5 n=15cells, VAMP1/2/3 n=35 cells, GM130 n=23 cells). **E.** SGC-CM obtained from SGC cultures treated with Brefeldin-A was analyzed by western blot for Fibulin-2. CD9, a marker of EV, is used as a control. Ponceau staining is shown for loading control. n= 3 independent experiments. **F.** SGC cell pellet obtained from SGC cultures treated with Brefeldin-A was analyzed by western blot for Fibulin-2. GAPDH is used as a loading control. Ponceau staining is shown as another loading control. n= 3 independent experiments. **G.** Quantification of Fibulin-2 level in SGC-CM shown in E. n=3 T-test; ***P < 0.001. **H.** Quantification of Fibulin-2 level in SGC cell pellet shown in F. n=3, T-test; *P < 0.05. **I.** EVs were isolated from SGC-CM prepared from cultures from WT or Fibulin2-KO mice and analyzed by western blot alongside the SGC lysate for Fibulin2, CD9 (EVs marker) and GM130 (Golgi-associated vesicles, negative control for EV). GAPDH is used as a loading control. n= 3 independent experiments.

To determine the ensemble of proteins secreted by SGCs, the SGC-conditioned media (SGC-CM) and the corresponding SGC cell pellet were collected at DIV8 after cultures were switched to serum-free media at DIV7, and analyzed by mass spectrometry following established protocols ^40–42^. Culture media alone was processed in parallel and served as a negative control. We identified 740 proteins in total, with 663 proteins present specifically in the SGC-CM and absent in culture media (Supplem. Data File 1). We performed GO enrichment analysis on SGC-secreted proteins, using proteins identified in the SGC cell pellet as a reference SGC proteome (Supplem. Data File 2). Using a cutoff of FDR < 0.5, we found that SGC secretory proteins were enriched for extracellular space and extracellular matrix (ECM) proteins (Fig. 1A). ECM proteins, collectively referred to as the matrisome, are divided into six categories: collagen, ECM glycoproteins, proteoglycans, ECM regulatory proteins, ECM-associated proteins, and secretory factors ^28,43^. We observed that the SGC-CM was enriched in ECM glycoproteins and ECM regulators (Fig. 1B). To further identify the SGCs-secreted proteins with cell-type-specific expression, we integrated the secretome data with our previously generated single cell RNAseq atlas of the mouse DRG ^39^. Proteins identified in SGCs-CM were filtered using stringent criteria (p < 0.05, fold-change > 2, minimal signal in negative controls), yielding 73 high-confidence secreted proteins, of which 71 had corresponding genes detected in the scRNAseq dataset. We then ranked candidates by absolute SGC expression level and by SGC specificity, defined as the expression ratio relative to the highest-expressing non-SGC cell type (Supplem. Data File 3). The top 4 candidates included *Me1*, *Dbi*, *Col28a1,* and *Fbln2*. While Me1 is not known to be a secreted protein, DBI secretion by cultured SGCs and its expression in SGCs *in vivo* have previously been demonstrated ^26^. Col28a1 is expressed in SGCs at the transcriptional level ^39,44^ and likely plays a role in ECM deposition around SGCs. Fibulin-2 (*Fbln2*) emerged as a top candidate for further investigation. Fibulin-2 is an ECM glycoprotein with roles in formation and stabilization of elastic fibers and basement membranes and also has diverse functions in the nervous system, such as spinal cord development ^33^, oligodendrocyte maturation ^36^, synapse formation ^34^ and neuron excitability in the spinal cord ^35^.

Fibulin-2 can be secreted via the canonical secretory pathway, and it can also be released by extracellular vesicles (EVs), such as astrocyte-derived exosomes ^34^. To investigate the mechanism by which Fibulin-2 is secreted, we first examined its subcellular localization in cultured SGCs. Fibulin-2 displayed partial overlap with VAMP1/2/3, and VAMP5, which all belong to the SNARE family of proteins, whose main function is to drive the fusion of vesicles with target membranes (Fig. 1C, D). While VAMP1/2/3 function in neurons and glial cells in the brain to regulate exocytosis ^45^, VAMP5 has been localized to multivesicular bodies in epithelial cells and suggested to mediate exosome release ^46,47^. Consistent with the possibility of Fibulin-2 being released by EVs, Fibulin-2 also co-localized with Lamp1 and CD63, markers of late endosomes and multivesicular bodies-like compartments, but not with the Golgi marker GM130 (Fig. 1C,D). To determine by which pathway SGCs secrete Fibulin-2, we treated cultured SGCs with the intracellular protein transport inhibitor Brefeldin A. We first confirmed the specificity of the Fibulin-2 antibody in western blots using lysate obtained wild-type (WT) or Fibulin-2 knock out (KO) mice ^48^. We observed that Fibulin-2 migrated at the expected molecular weight of ~135 kDa, and that the antibody recognizes a non-specific band at ~150 kDa in both DRG and SGC lysates but not in the SGC-CM (Fig. S1E,F). We noted that in the SGC-CM, Fibulin-2 migrated at a slightly higher molecular weight compared to DRG and SGC lysate. When SGC cultures were treated with Brefeldin A, Fibulin-2 secretion was decreased and the level of Fibulin-2 in the SGCs lysate was increased (Fig 1E,H). To directly examined whether Fibulin-2 is secreted via EVs, we isolated EVs from SGC-CM prepared from WT or Fibulin-2 KO SGC cultures. Western blot analysis showed that purified EVs were positive for the common EV marker CD9 and negative for the Golgi marker GM130 (Fig 1I, S1E) as expected, and Fibulin-2 was detected in EVs lysate from WT but not Fibulin-2 KO (Fig. 1I, S1E). Together, these results suggest that cultured SGCs secrete Fibulin-2 via both the classical secretory pathway and the EVs-mediated pathway.

### SGCs expressing Fibulin-2 ensheathe diverse subtypes of sensory neurons

**Figure 2.**
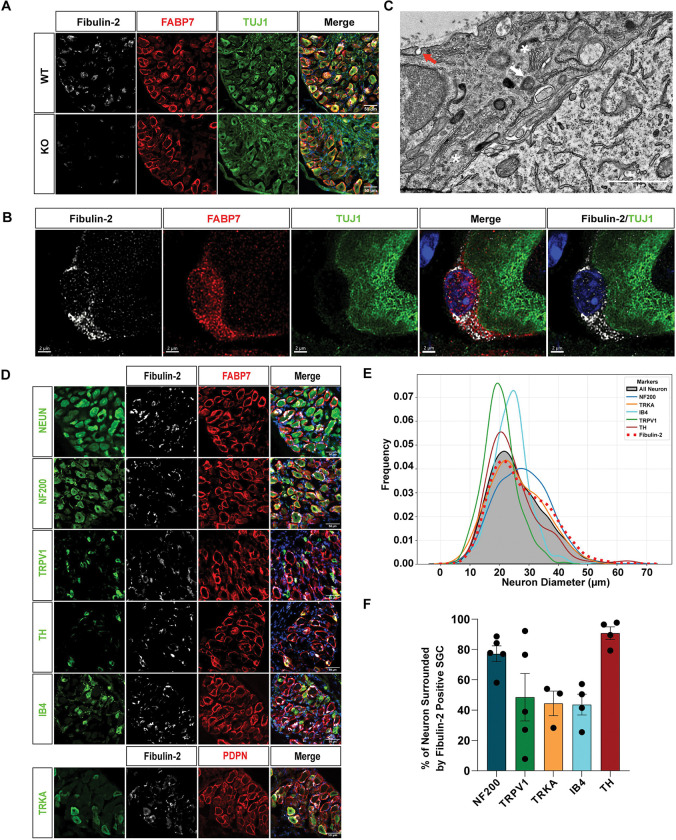
SGCs expressing Fibulin-2 ensheathe diverse subtypes of sensory neurons **A.** Representative images of DRG sections from WT and Fibulin-2 KO mice immunostained for Fibulin-2, FABP7, TUJ1, and DAPI. Scale bar 50μm. Two DRG sections from n=3 mice were imaged for each group. **B.** Super-resolution imaging of DRG tissue section highlighting the subcellular localization of Fibulin-2 in SGCs near the perinuclear region. Fibulin-2 (Red), FABP7 (White), TUJ1 (Green), and DAPI (Blue). Scale bar 2μm **C.** Electron micrograph of DRG sections showing the portion of an SGC covering the neuron soma. The SGC cytoplasm contains multivesicular bodies (white arrow), vesicle fusion/budding at the plasma membrane (red arrow), and a Golgi apparatus (asterisk). Scale bar 1μm **D.** Immunofluorescence of DRG section showing localization of Fibulin-2 in SGCs (labeled for Fabp7 or PDPN) surrounding neurons labeled with NeuN, NF200, TRPV1, TH, TRKA, and IB4. Scale bar 50μm. **E.** Quantification of the size distribution of the indicated DRG neurons subtypes. The size of neurons surrounded by Fibulin-2 positive SGC is indicated by a red dotted line. A total of 8956 neurons were analyzed from n=7 mice, with three DRG sections per mice. **F.** Quantification of the percentage of neuronal subtype surrounded by Fibulin-2-positive SGCs. Each data point in the bar graph represents an independent biological replicate. NF200 (n=5 mice), TRPV1 (n=5 mice), TRKA (n=3 mice), IB4 (n=4 mice), TH (n=4). Two sections from each biological replicate were imaged and used for quantification. Error bars represent SEM.

We next determined the localization of Fibulin-2 *in vivo* in DRG sections. Fibulin-2 was localized in FABP7-positive SGCs surrounding neuronal soma labeled with TUJ1 in wild-type mice and no Fibulin-2 signal was detected in DRG sections from Fibulin-2 KO mice, as expected (Fig 2A). Super resolution imaging revealed that Fibulin-2 was enriched near the perinuclear region in SGCs (Fig. 2B). Electron microscopy imaging revealed that the perinuclear region of SGCs is enriched with Golgi apparatus and Golgi-derived vesicles (Fig. 2C), as also previously determined ^16^. Occasionally, we observed structures resembling multivesicular bodies and vesicular budding or fusion events in the plasma membrane facing both the neuron or the extracellular space (Fig. 2C), suggesting that SGCs *in vivo* can secrete Fibulin-2 towards the neuron and the DRG parenchyma.

We noted that unlike FABP7, Fibulin-2 was not detected as a ring pattern around the neuron soma. This could result from Fibulin-2 enrichment in the perinuclear region or to selective expression of Fibulin-2 around neuronal subtypes. Indeed, DRG neuron subtypes can be distinguished by their molecular, morphological, and physiological properties, which affect their responses to various stimuli such as pain, touch, pressure, and temperature ^49^. We thus investigated whether Fibulin-2 is enriched in SGCs that ensheathe specific subtypes of neurons in the DRG. We first utilized Cellpose to identify the neuron soma stained with the pan-neuronal marker NeuN and measured the size of the neurons using ImageJ. This analysis revealed that Fibulin-2-positive SGCs encircled neurons of all sizes (Fig. 2D, E). We next employed different neuronal markers, including NF200 for large-diameter neurons, IB4 for small-diameter non-peptidergic neurons, TrkA for nociceptors and thermoreceptors, Trpv1 for heat-sensing nociceptors, and tyrosine hydroxylase (TH) for C-low threshold mechanoreceptors (Fig. 2D). We quantified the percentage of each sensory neuron type that was surrounded by Fibulin-2 positive SGCs. We found that a vast majority of TH-positive neurons and NF200-positive neurons were surrounded by Fibulin-2 positive SGCs, as well as over 40% of the small diameter nociceptors and thermoreceptors (Fig. 2F). These results suggest that Fibulin-2-positive SGCs are not restricted to any specific subtype of DRG neurons and may play a role in regulating the functions of a diverse range of neuronal subtypes, including mechanoreceptors, nociceptors, and thermoreceptors.

### Fibulin-2 regulates excitability of sensory neurons

In the spinal cord, Fibulin-2 acts on GABA_B_ receptors (GABA_B_Rs) to suppress their activation, effectively reducing GABA_B_R-mediated inhibition, and increasing excitability ^35^. GABA_B_Rs are also expressed in the soma of some sensory neurons ^50,51^ Recent studies have established that somatic excitability is critical in sensory information transmission ^52,53^. In addition, somatic hyperexcitability in nociceptors has been shown to play a role in the priming of certain pain states ^54,55^ and persistent nociceptor somatic hyperactivity has been suggested to drive painful hypervigilance during persistent physical impairment ^55^. Moreover, somatic firing patterns and excitability profiles of human sensory neurons correlate closely with the pain history of the donor ^56^. Together, these studies emphasize the importance of somatic excitability in sensory processing and various pain states.

**Figure 3.**
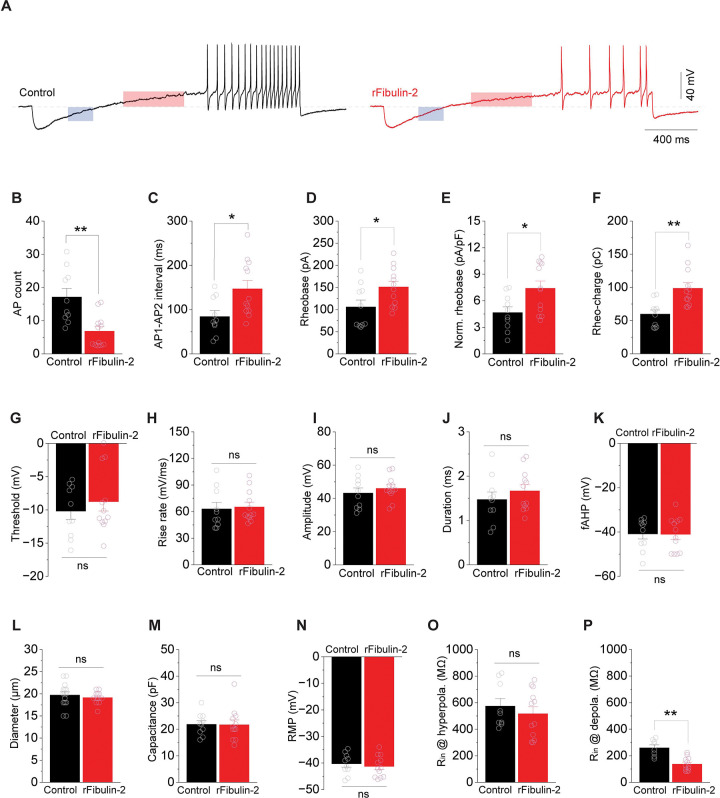
Fibulin-2 regulates the excitability of DRG neurons. **A**. Sample traces of action potentials (APs) recorded from Control and rFibulin-2-treated DRG neurons. APs were evoked by ramp current injection (0.15 pA/ms) via recording pipettes. Traces within shaded areas were used to calculate input resistance at hyperpolarization (blue, summarized in **O**) and depolarization (red, summarized in **P**) states. **B-F**. rFibulin-2 treatment decreased excitability of DRG neurons, as shown by reduced number of APs (**B**), and increases in the initial inter-AP interval (**C**), AP rheobase (**D**), normalized rheobase (**E**), and rheobase charge transfer (**F**). Number of cells tested from 3 independent experiments: control n = 10; rFibulin-2: n = 12. **G-K**. rFibulin-2 did not affect multiple other AP parameters, including AP threshold (**G**), maximal rise rate (**H**), amplitude (**I**), duration (**J**) and fast afterhyperpolarization (fAHP) (**K**). Number of cells tested from 3 independent experiments: control n = 10; rFibulin-2: n = 12. **L-N**. The recorded cells have comparable size (**L**), membrane capacitance (**M**), and resting membrane potential (RMP) (**N**). Number of cells tested from 3 independent experiments: control n = 10–15; rFibulin-2: n = 12–13. **O-P**. rFibulin-2 decreased input resistance of DRG neurons at depolarization state (**P**). However, it did not affect the input resistance at hyperpolarization state (**O**). Number of cells tested from 3 independent experiments: control n = 8–9; rFibulin-2: n = 12. T-test; *P < 0.05; **P < 0.01; ns, not significant.

Therefore, we asked whether Fibulin-2 plays a role in regulating somatic excitability of small/medium DRG sensory neurons, following a protocol we previously described ^19,57^. Specifically, we performed whole-cell recordings in short-term DRG cultures treated (or not) with 2μg/mL of recombinant human Fibulin-2 for 24 hours (Fig. 3A). This concentration is similar to what was used to measure Fibulin-2 effect on GABA_B_R ^35^. Human Fibulin-2 is highly conserved across species, with the human protein sharing 82% amino acid identity compared to both mouse and rat. Action Potentials (APs) were evoked by a ramp-current injection (ramp rate 0.15 pA/ms). We found that Fibulin-2 treatment significantly decreased the number of AP fired, and it also prolonged the interval between the 1st and 2nd APs (Fig. 3A-C). These results suggest that Fibulin-2 operates in DRG via a mechanism that is distinct from what has been observed in the spinal cord ^35^, decreasing the excitability of DRG neurons. To probe how Fibulin-2 regulates neuronal excitability, we examined several AP parameters. Only the 1st APs in the train were included in this analysis to avoid AP parameters being affected by cumulative inactivation of Na^+^ and K^+^ channels in subsequent APs. We found that Fibulin-2 significantly increased current threshold – the rheobase (Fig. 3D-F), but did not affect voltage threshold, maximal AP rise rate, AP amplitude, duration or fast afterhyperpolarization (fAHP) (Fig. 3G-K).

The observation that Fibulin-2 changes rheobase without affecting voltage threshold can be attributed to alterations in cell size/capacitance, resting membrane potential (RMP), or membrane input resistance. Since the cells we recorded had comparable size, capacitance and RMP (Fig. 3L-N), we measured the input resistance at hyperpolarization and depolarization states from the ramp-evoked AP traces (Fig. 3A, **shaded areas**). For both control and Fibulin-2-treated cells, the input resistance at depolarization state (Fig. 3P) was markedly lower than that of hyperpolarization state (Fig. 3O), which is likely due to activation of voltage-dependent K^+^ conductances. Importantly, Fibulin-2 treatment significantly reduced the input resistance at depolarization state (Fig. 3P), without alterations of input resistance at hyperpolarization state (Fig. 3O). Together with the above effects of Fibulin-2 on increasing rheobase without altering the RMP and threshold, these results suggest that Fibulin-2 acts by enhancing a voltage-dependent K^+^ conductance to reduce input resistance in response to membrane depolarization. It is worth mentioning that Fibulin-2 regulation of excitability is unlikely to be mediated by Na^+^ channels, because it did not change voltage threshold, maximal AP rise rate, or AP amplitude in DRG neurons.

### Fibulin-2 increases voltage-dependent K^+^ conductance (I_A_ currents) mediated by Kv4 channels in DRG neurons

**Figure 4.**
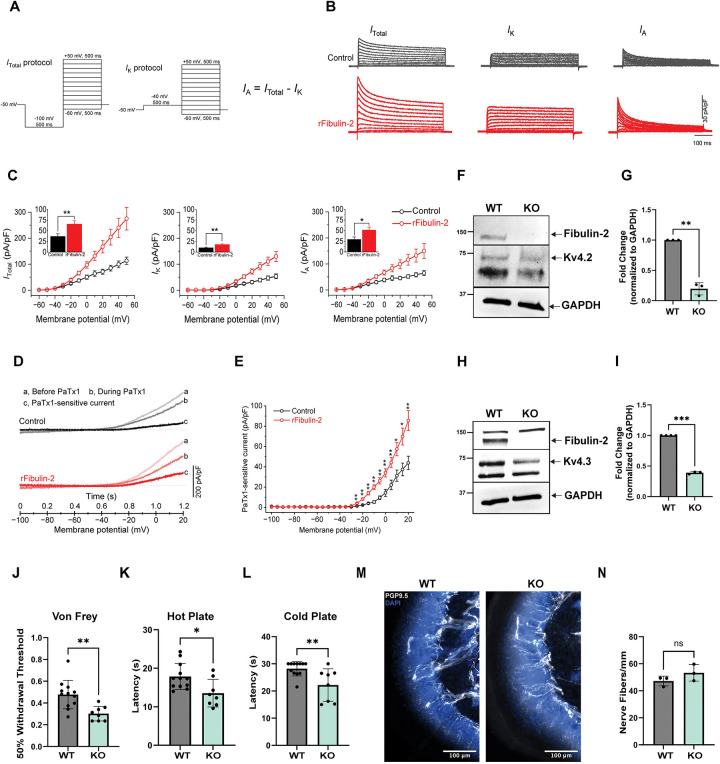
Kv4 channels contribute to Fibulin-2 regulation of excitability in DRG neurons. **A.** Voltage protocols for measurement of different types of K^+^ currents: total (*I*_Total_), K-type (*I*_K_) and A-type (*I*_A_) K^+^ currents. **B.** Sample traces of voltage-dependent K^+^ currents *I*_total_ (left), *I*_K_ (middle) and *I*_A_ (right) evoked by the protocols in (**A**) from Control (upper panel) and rFibulin-2 treated DRG cells (lower panel). **C.** rFibulin-2 increases voltage-dependent K^+^ currents *I*_Total_ (left), *I*_K_ (middle) and *I*_A_ (right) in DRG cells. Insert bar graphs are K^+^ currents at membrane potential of −10 mV (around voltage threshold level), indicating that rFibulin-2 decreases excitability mainly mediated by enhancement of *I*_A_ conductance, which reduces input resistance. Number of cells tested from 3 independent experiments: control n = 10; rFibulin-2: n = 8. **D.** Phrixotoxin-1 (PaTx1) was used to isolate Kv4 current evoked by voltage ramp (−100 to +20 mV, 100 mV/s). Sample traces of ramp-evoked K^+^ currents before (a) and during (b) application of PaTx1, and the PaTx1-sensitive current (c, c = a - b). Currents were normalized to membrane capacitance for better comparison. **E.** I-V curves were constructed from the ramp-evoked Kv4 current (mean current value over 0.1 mV intervals from averages of five trials for each cell to approximate quasi-steady-state current). Note PaTx1 significantly increases the Kv4 current when the membrane potentials are depolarized to positive values greater than −25 mV. Number of cells tested from 3 independent experiments: control n = 6; rFibulin-2: n = 6; T-test; *P < 0.05; **P < 0.01. **F.** Representative western blot of control and Fibulin-2 KO DRG lysate analyzed for Fibulin-2 and Kv4.2. GAPDH is used as a loading control. **G.** Quantification of Kv4.2 expression in control and Fibulin-2 KO mice. n=3 WT and n=3 Fibulin-2 KO mice. T-test; **P < 0.01. **H.** Representative western blot of control and Fibulin-2 KO DRG lysate analyzed for Fibulin-2 and Kv4.3. GAPDH is used as a loading control. **I.** Quantification of Kv4.3 expression in control and Fibulin-2 KO mice. n=3 WT and n=3 Fibulin-2 KO mice. T-test; ***P < 0.001 **J.** Fibulin-2 KO mice show hypersensitivity to mechanical stimuli compared to controls, measured by the Von Frey Test. 12 WT and 8 Fibulin-2 KO mice were used. Two-Way Anova. *p < 0.05, **p < 0.01, ***p<0.001. **K.** Fibulin-2 KO mice exhibit hypersensitivity to heat stimuli compared to controls, measured by the Hot-Plate test. 12 WT and 8 Fibulin-2 KO mice were used. Two-Way Anova. *p < 0.05, **p < 0.01, ***p<0.001. **L.** Fibulin-2 KO mice exhibit hypersensitivity to cold stimuli compared to controls, measured by the Cold-Plate test. 12 WT and 8 Fibulin-2 KO mice were used. Two-Way Anova. *p < 0.05, **p < 0.01, ***p<0.001. **M.** Representative immunofluorescence images of the hindpaw of control and Fibulin-2 KO mice immunostained for PGP9.5 (white) and DAPI (blue). Three sections from n=3 mouse per group were used. **N.** Quantification of intraepidermal nerve fiber density (IENFD). n=3 mice per genotype. T-test, ns- non-significant

Voltage-dependent K^+^ channels (Kv) are crucial in controlling neuronal excitability by regulating membrane potential, input resistance, rheobase, and firing frequency ^58^. In small/medium DRG sensory neurons, there are two major types of voltage-dependent K^+^ currents: sustained delayed rectifier K^+^ current (I_K_) and transient A-type K^+^ current (I_A_) ^59^. We thus examined whether the decreased excitability caused by Fibulin-2 was due to alterations in the K^+^ currents. The total K^+^ current (I_Total_) was evoked by a series of 500-ms test pulses from −60 to +50 mV with a 10-mV increment, preceded by a prepulse of −100 mV for 500 ms (Fig. 4A, **left**). I_K_ was isolated by replacing the prepulse command potential of −100 mV with −40 mV (Fig. 4A, **middle**), which inactivates the I_A_ component. I_A_ was then obtained by subtraction of I_K_ from I_Total_. For better comparison among cells, we normalized the current to the cell capacitance. As expected, Fibulin-2 treatment significantly increased the K^+^ currents I_Total_, I_K_ and I_A_ at membrane potentials above −20 mV (Fig. 4B,C), consistent with the observation above that Fibulin-2 only affected input resistance in depolarization state. Notably, at the membrane potential around the threshold level (about −10 mV), at which the active K^+^ currents play key roles in controlling AP initiation, the increase of I_A_ contributes most (~76%) of the increase in total K^+^ current (Fig. 4C).

In DRG neurons, I_A_ can originate from Kv channels encoded by several molecular entities, including Kv1.4, Kv4.2 and Kv4.3 ^59^. In our culture conditions, the majority of the neurons we recorded were from small/medium diameter cells ^19,57^, in which I_A_ is known to be mediated predominately by Kv4 channels ^60,61^. We therefore asked whether Kv4 channels are involved in the Fibulin-2 regulation of neuronal excitability. Using Phrixotoxin-1 (PaTx1, 50 nM), a potent and selective Kv4.2 and Kv4.3 blocker, we isolated a PaTx1-senstive component of I_A_ current evoked by a voltage ramp protocol, as previously described ^62^. We found that Fibulin-2 significantly increased PaTx1-sensitive current when the membrane potential was above −25 mV (Figure 4D,E). These results indicate that Kv4 channels contribute to Fibulin-2 enhancement of I_A_, leading to a reduction in membrane input resistance and an increase in rheobase, subsequently decreasing excitability and AP firing in small/medium DRG neurons.

### Fibulin-2 KO mice exhibit hypersensitivity to thermal and mechanical stimuli

Reduced expression of Kv4.2 and Kv4.3 in neuropathic pain model induces hypersensitivity, a major symptom of neuropathic pain ^63–65^. Using single cell RNAseq atlas of the mouse DRG ^39^, we found that Kv4.3/*Kcnd3* is expressed at the transcript level in a wider range of neurons compared to Kv4.2/*Kcnd2*, and it is highly enriched in mechanical- and pain sensing neurons (Fig S2A). Based on the above observations, we hypothesized that the absence of Fibulin-2 would lead to decreased Kv4 levels in DRG neurons of Fibulin-2 KO mice and increased mechanical and thermal sensitivity. To test this hypothesis, we first measured the levels of Kv4.2 and Kv4.3 in the DRG of WT and Fibulin-2 KO mice. We observed a small but significant decrease in Kv4.2 and Kv4.3 protein expression in the DRG of Fibulin-2 KO mice compared to control mice (Fig. 4F-I). We then conducted behavioral sensitivity tests, utilizing Von Frey for mechanical sensitivity, Hot Plate and Cold Plate for thermal sensitivity. Our results indicated that Fibulin-2 KO mice exhibited hypersensitivity to both mechanical and thermal stimuli (Fig. 4J-L). This hypersensitivity is unlikely due to alteration in skin innervation, as Fibulin-2 KO mice exhibit no major changes in the skin ^48^, and we did not detect any changes in intraepidermal nerve fiber density (IENFD) (Fig. 4M,N). These results indicate that Fibulin-2 KO mice are sensitive to mechanical and thermal stimuli, which is in part mediated by a decrease in the levels of Kv4.3 and Kv4.2 in DRG neurons.

## DISCUSSION

Our study identifies SGCs as a critical source of the extracellular matrix glycoprotein Fibulin-2 in DRG, revealing a novel mechanism by which SGCs modulate sensory neuron excitability and pain responses. Through proteomic profiling, we demonstrate that SGCs secrete Fibulin-2, which, when applied to cultured sensory neurons, reduces neuronal excitability by enhancing Kv4-mediated potassium current I_*A*_. This effect is further substantiated by behavioral assays in Fibulin-2 KO mice, which exhibit lower levels of Kv4 channels and hypersensitivity to thermal and mechanical stimuli, paralleling reduced Kv4.3 expression in DRG neurons after nerve injury ^63–67^.

SGCs are increasingly recognized as dynamic regulators of sensory neuron excitability and pain, acting through a complex network of molecular and cellular interactions ^7,9,68–70^. Our findings expand the understanding of SGC-neuron communication, highlighting SGC-derived Fibulin-2 as a key regulator of neuronal activity. Previous studies have implicated SGCs in pain modulation through various mechanisms, including SGGs’ role in potassium buffering via Kir4.1 channels, gap junction coupling and purinergic signaling ^7,9,68–70^. Activated SGCs also release a variety of pro-inflammatory cytokines ^71–73^. However, these experiments were done with mixed DRG culture or DRG explants and thus the measured release of a given molecule cannot be affirmatively attributed to SGCs. These cytokines act on neurons to increase their excitability and firing rates, contributing to both acute and chronic pain states ^7,9,68–73^. Whether SGCs can reduce neuronal excitability is less well established. A recent study has demonstrated that SGCs secrete DBI ^26^ and increasing DBI levels using viral approaches reduces sensitivity to mechanical stimulation and alleviates mechanical allodynia in neuropathic and inflammatory pain models. Conversely, reducing DBI levels in SGCs results in robust mechanical hypersensitivity with no major effects on other sensory modalities. These effects of DBI are mediated by its actions as an endogenous unconventional agonist and positive allosteric modulator of GABA_A_ receptors in the DRG ^26^. In the spinal cord, Fibulin-2 interacts with presynaptic GABA_B_Rs, including those on nociceptive afferents ^35^, and inhibits GABA_B_R activation, presumably by disrupting agonist-induced conformational changes ^35^, thereby increasing afferent excitability. Although GABA_B_Rs have also been found in the soma of some DRG neurons ^74^, Fibulin-2 effects on somatic DRG neuron excitability cannot be explained by this mechanism since Fibulin-2 acting by suppressing GABA_B_R activation would have resulted in opposite effects on excitability and K^+^ currents than what we observed. Indeed, our data suggest that Fibulin-2 acts on the soma of DRG neurons via another mechanism, in which it enhances Kv4-mediated I_A_ currents to reduce neuronal excitability and alter sensitivity to pain stimuli. Together, our data identified Fibulin-2 as an SGC-secreted factor that adds a new dimension to the molecular landscape governing sensory neuron excitability and pain perception. Whether *in vivo*, Fibulin-2 is released constitutively or whether its secretion is context dependent will require further investigation.

Fibulin-2’s modulation of Kv4.2 and Kv4.3 channels is particularly noteworthy. Kv4 channels are known to mediate subthreshold I_A_ currents, which are crucial for controlling AP initiation and firing frequency in small/medium diameter nociceptors. Our data suggest that Fibulin-2 modulate the expression of these channels, thereby regulating neuronal excitability. The decrease in Kv4.3 expression in Fibulin-2 KO mice and the resultant hypersensitivity to mechanical and thermal stimuli underscore the physiological relevance of this pathway. Whether Fibulin-2 regulates Kv4 channels via increased transcription, translation, or regulates the surface expression of the channels remains to be tested. In other systems, Fibulin-2 was shown to function upstream of several signaling pathways, including TGF-β/Smad2, Notch, Ras-MEK-ERK1/2, Wnt/beta-catenin and integrin ^22,36,75^. The precise role of Fibulin-2 in regulating these pathways is highly dependent on the specific tissue type, disease context, and cellular microenvironment. While ERK1/2 and Notch signaling pathways are known to regulate Kv4 channel function ^76–78^, whether Fibulin-2’s effects on Kv4.2 and Kv4.3 expression are dependent on ERK1/2 activation remains to be determined. Emerging evidence also indicates that Fibulin-2 can be cleaved by ADAMTS-5, with ADAMTS-12 acting as an inhibitor of Fibulin-2 cleavage in breast cancer models ^79^. The presence of ADAMTS-5 in SGCs and fibroblasts within the DRG observed at the transcriptional level ^39^ raises the possibility that Fibulin-2 proteolysis may dynamically regulate the cellular microenvironment. This may not only impact neuronal excitability but also potentially influence the migratory properties of glial and immune cells. This proteolytic regulation could have broader implications for pain states and tissue remodeling following injury.

Fibulin-2 interacts with various extracellular matrix proteins, including brevican ^80^, which is highly enriched in SGCs in the DRG ^19,39,44^ indicating possible autocrine roles. Other partners like versican and fibrillin-1 are present in fibroblasts, and nidogen in both fibroblasts and SGCs ^39^, highlighting the potential for Fibulin-2 to influence the DRG microenvironment through autocrine and paracrine mechanisms. Additionally, our findings show that SGCs also secrete Fibulin-5, though at lower levels than Fibulin-2. Given that Fibulin-2 and Fibulin-5 both contribute to tissue integrity and nerve function ^81–84^, their interplay may further modulate sensory neuron activity in the DRG. Together, these interactions support our results that SGC-derived Fibulin-2 regulates neuronal excitability by shaping the extracellular environment and may work in concert with other members of the fibulin family of proteins.

In summary, our work positions SGC-secreted Fibulin-2 as a pivotal regulator of sensory neuron excitability, acting through modulation of Kv4 channels, opening new avenues for therapeutic intervention in chronic pain conditions. Targeting Fibulin-2 or its downstream pathways could provide a novel strategy for pain management, potentially overcoming the limitations of current treatments that focus primarily on sensory neurons and often result in suboptimal analgesia and adverse effects. Extending these findings to human DRG tissue and pain models will be essential for assessing the clinical relevance of SGC-derived Fibulin-2.

## Materials and Methods

### Experimental Animals

All mice were approved by the Washington University School of Medicine Institutional Animal Care and Use Committee (IACUC) under protocol A3381–01. All experiments were performed in accordance with the relevant guidelines and regulations. All experimental protocols involving mice were approved by Washington University School of Medicine (protocol # 24–0078). Mice were housed and cared for in the Washington University School of Medicine animal care facility. This facility is accredited by the Association for Assessment & Accreditation of Laboratory Animal Care (AALAC) and conforms to the PHS guidelines for Animal Care. Accreditation - 7/18/97, USDA Accreditation: Registration # 43-R-008.

Mice of different age groups were included in the study, specifically 2–3 months-old (adult, female and male). Wild-type C57BL/6 mice were purchased from Envigo (Envigo #027) and Jackson Laboratory (Stock No: 000664). Fibulin-2 KO mice (B6.129S1-Fbln2tm1Chu/J) were obtained from The Jackson Laboratory. Mice were housed in the animal facility at Washington University in St. Louis, where temperature (64–79 °F) and humidity (30%–70%) were carefully controlled. They were socially housed in individually ventilated cages, with 1–5 mice per cage, and subjected to a 12 hr light/dark cycle (6 am/6 pm). Mice had unrestricted access to food and water throughout the study.

### SGCs primary culture

The protocol for the primary culture of SGCs was adapted from a previous study ^38^. In brief, L3-L5 dorsal root ganglia (DRGs) were isolated from both sides of the mice using dissociation media consisting of Hank’s Balanced Salt Solution (HBSS) supplemented with 1M Hepes. The DRGs were incubated in a Papain solution, which contained 40 U of Papain, NaOH, DNase I, and L-cysteine, for 20 minutes at 37 °C. Following this, the samples were digested with Collagenase for an additional 20 minutes at 37 °C. After performing two washes with complete DMEM media (Gibco Catalog# 11054020), 200nM L-Glutamine, 10% FBS), the DRG samples were triturated to create a single-cell suspension. This suspension was then passed through a 50 μm filter, followed by a 10 μm filter to ensure cell quality. Subsequently, the SGC cells were seeded in complete DMEM media (DMEM supplemented with 10% FBS) and incubated at 37 °C. After 24 hours, the media were replaced with fresh complete DMEM and the culture media was changed every two days.

For western blot, the SGCs-CM was collected after 8 days in vitro (DIV8). The conditioned media was centrifuged at maximum speed for 30 minutes to eliminate any cell debris. The cell-free SGCs-CM was then filtered using an Amicon filter with a cut-off of 3 kDa (Sigma Catalog# UFC200324) and the concentrated SGCs-CM was stored at −80 °C.

For mass spectrometry analysis, the cells were cultured for 7 days in complete DMEM. At DIV7, the culture medium was switched to DMEM without FBS. The SGCs-CM was collected at DIV8 and centrifuged at 2000g for 10 minutes to remove any cell debris. The resulting conditioned media, along with a blank control sample, were then flash-frozen and stored at −80 °C.

For drug treatments, SGCs were cultured for 10 days to achieve a confluency of 80% in complete DMEM media. The SGCs were then treated with 3 μg/ml Brefeldin-A (Invitrogen, 4506–51) for 24 hours. After 24h, SGCs-CM and SGCs pellets were collected and processed for Western blot.

For bulk RNA-seq, SGCs were cultured for 7 days in complete DMEM media. After 7 days, culture medium was switched to DMEM medium with or without FBS and incubated for 24 h. At DIV8, RNA was extracted from cultured SGCs using the RNAeasy Micro Kit (QIAGEN, Cat# 74004), flash-frozen and stored at −80 °C.

### RNA sequencing

Total RNA integrity was determined using Agilent Bioanalyzer or 4200 Tapestation. Library preparation was performed with 5 to 10ug of total RNA with a Bioanalyzer RIN score greater than 8.0. Ribosomal RNA was removed by poly-A selection using Oligo-dT beads (mRNA Direct kit, Life Technologies). mRNA was then fragmented in reverse transcriptase buffer and heating to 94 degrees for 8 minutes. mRNA was reverse transcribed to yield cDNA using SuperScript III RT enzyme (Life Technologies, per manufacturer’s instructions) and random hexamers. A second strand reaction was performed to yield ds-cDNA. cDNA was blunt ended, had an A base added to the 3’ ends, and then had Illumina sequencing adapters ligated to the ends. Ligated fragments were then amplified for 12–15 cycles using primers incorporating unique dual index tags. Fragments were sequenced on an Illumina NovaSeq X Plus using paired end reads extending 150 bases. Basecalls and demultiplexing were performed with Illumina’s bcl2fastq software with a maximum of one mismatch in the indexing read. RNA-seq reads were then aligned to the Ensembl GRCm39.113 primary assembly with STAR version 2.7.11b. Gene counts were derived from the number of uniquely aligned unambiguous fragments by Subread:featureCount version 2.0.8.

### RNA sequencing analysis

Gene counts were normalized to counts per million (CPM) using edgeR (v4.0.16). To assess the cellular identity of cultured SGCs, bulk RNA-seq expression profiles were compared to pseudobulk profiles derived from our previously described single-cell RNA-seq atlas of the mouse DRG ^39^. Single-cell data were aggregated by major cell class (glia, neurons, immune cells, endothelial cells, erythrocytes, fibroblasts, and mural cells), and the top 5,000 highly variable genes shared between platforms were identified using Seurat (v5.1.0). Bulk and pseudobulk count matrices were jointly normalized using the trimmed mean of M-values (TMM) method, converted to log-CPM, and transcriptional similarity was quantified by Spearman correlation. To identify SGC-enriched secreted proteins, candidates from the mass spectrometry analysis were cross-referenced with the single-cell atlas. Expression was quantified across cell type clusters, and candidates were ranked by both absolute SGC expression level and SGC specificity, defined as the ratio of SGC expression to the highest-expressing non-SGC cell type. Gene expression across neuronal subtypes was visualized using the single-cell data. All analyses were performed in R (v4.3.3).

### Real-time PCR

For quantitative real-time PCR, RNA was isolated from SGC cells using the RNAeasy Micro Kit (QIAGEN, Cat# 74004). For cDNA synthesis, 500 ng of RNA was used and converted into cDNA with the High-Capacity cDNA Reverse Transcription Kit (ThermoFisher, Catalog: 4368814), following the manufacturer’s protocol. Quantitative real-time PCR was set up using the SYBR^™^ Green Universal Master Mix (Applied Biosystem Cat# 4309155) and gene-specific primers on the QuantStudio 6 Flex system. The expression fold change of each gene was calculated using the ΔCT method, with GAPDH serving as the normalization control. The researcher was not blinded during the analysis.

### Western blot

DRG samples were collected from mice and lysed using RIPA buffer (Cell Signaling, catalog #9806). Protein concentrations were measured using the BCA kit (ThermoFisher, Pierce^™^ Catalog #23227). The samples were prepared in Lamellae buffer and heated at 95 °C for 10 minutes to denature the proteins. They were then loaded into a precast gel from Bio-Rad and transferred to a PVDF membrane (Bio-Rad Catalog# 1620177). To stain for total protein, the PVDF membrane was incubated with Ponceau stain for 2 minutes, and an image was captured. After staining, the membrane was washed with PBST (1X PBS containing 0.1% Tween-20). The membrane was blocked with 3% BSA for 1 hour at room temperature, followed by incubation with the primary antibody overnight at 4 °C. After three washes with PBST, the membrane was incubated with an HRP-conjugated secondary antibody for 1 hour at room temperature and washed three more times with PBST. Blots were developed using the chemiluminescence method with the Thermo Signal Dura and imaged on Jess Automated Western Blot System.

### Mass spectrometry

Samples were concentrated using the 3 kDa MWCO filter and subsequently purified by acetone/trichloroacetic acid precipitation. The resulting protein pellet was resuspended in 8M urea and 0.4 M ammonium bicarbonate, reduced with 4mM dithiothreitol, and alkylated with 18 mM iodoacetamide. One ug of trypsin was added prior to an overnight incubation at 37°C. The resulting peptides were desalted using the C18 spin column and dried peptide was reconstituted in 0.1% formic acid and loaded onto a Neo trap cartridge coupled with an analytical column (PepMap^™^ Neo C18, 75 μm × 50 cm) over 120 min linear gradient from 2% to 35% of solvent B (0.1% formic acid in acetonitrile) using a Vanquish Neo UHPLC System coupled to an Orbitrap Eclipse Tribrid Mass Spectrometer with FAIMS Pro Duo interface (Thermo Fisher Scientific). Data were searched using Mascot (v.3.1 Matrix Science) against the mouse SwissProt database. Trypsin was selected as the enzyme, and the maximum number of missed cleavages was set to 3. The precursor mass tolerance was set to 10 ppm, and the fragment mass tolerance was set to 0.6 Da for the MS2 spectra. Carbamidomethylated cysteine was set as a static modification, and dynamic modifications were set as oxidized methionine, deaminated asparagine/glutamine, and Protein N-terminal acetylation. The search results were validated with 1% FDR protein threshold and a 90% peptide threshold using Scaffold (v5.3.0 Proteome Software).

### Immunofluorescence

Dorsal root ganglia (DRGs) from L3 to L5 were harvested from the mice and fixed in 4% paraformaldehyde (PFA). After fixation, the DRG samples were transferred to a 30% sucrose solution prepared with 1x PBS and incubated overnight at 4 °C. The samples were subsequently embedded in optimal cutting temperature (OCT) compound and stored at −20 °C for further processing. The frozen DRG samples were sectioned at a thickness of 10 μm using a cryostat and maintained at −20 °C until needed. Before staining, the sections were removed from −20 °C and allowed to acclimate at room temperature for 30 minutes. They were then washed twice with 1x PBS for 5 minutes each. Next, the sections were treated with a blocking solution containing 3% bovine serum albumin (BSA) and 0.3% Triton X-100 in PBS for 1 hour at room temperature. Following the blocking step, the sections were incubated overnight at 4 °C with the primary antibody diluted in 1.5% BSA and 0.3% Triton X-100 in PBS. The sections were washed three times with 1x PBS, again for 5 minutes each. After washing, the samples were incubated for 1 hour at room temperature with an Alexa Fluor-conjugated secondary antibody, diluted in 1.5% BSA and 0.3% Triton X-100 in PBS. Following this incubation, the samples were treated with 300 nM 4′,6-diamidino-2-phenylindole (DAPI, Sigma-Aldrich, Catalog# D9542) for 10 minutes. The samples were washed twice with 1x PBS and finally mounted using Prolong Gold Antifade mounting reagent.

For intraepidermal nerve fiber density measurements, fixed, free-floating 50-μm-thick sections of footpads from the hind limbs were stained with rabbit anti-Protein Gene Product 9.5 (1:500, LSBIO, Cat# LS-B5981–50), followed by AF594-conjugated secondary antibody (1:500, Invitrogen, Cat# A-21207). Sections were mounted and coverslipped using VECTASHIELD anti-fade mounting media with DAPI (Vector Laboratory, Cat# H-2000). PGP 9.5 positive intraepidermal nerve fibers (IENFs) crossing into the epidermis were counted from Z-stack images. IENFD densities were averaged from three to four sections for each animal.

All images were acquired using a Nikon Ti2 fluorescent microscope with a 20x objective. For the localization of Fibulin-2 in DRG sections, images were acquired using a Nikon W1 SoRa confocal microscope under a 60x objective. For super-resolution imaging, Nikon AX-R with NSPARC super-resolution confocal microscope was used with a 60X objective.

### Electron microscopy

For transmission electron microscopy, mice were perfused with 2.5% glutaraldehyde and 4% paraformaldehyde in 0.1M Cacodylate buffer, followed by post fix. A secondary fix was done with 1% osmium tetroxide. The tissue was dehydrated with ethanol and embedded with spurr’s resin. Thin sections (70 nm) were mounted on mesh grids and stained with 8% uranyl acetate followed by Sato’s lead stain. Sections were imaged on a Jeol (JEM-1400) electron microscope and acquired with an AMT V601 digital camera at the Washington University Center for Cellular Imaging.

### Adult DRG culture

L3 to L5 dorsal root ganglia (DRGs) were collected from both sides of the mice and placed in cold dissection media (HBSS (Gibco; Catalog#: 14175–079) supplemented with 10% 1 M Hepes (Corning Catalog# 25–060-CI). The DRGs were then incubated in a Papain solution containing 15U/mL Papain (Worthington Biochemical; Catalog#: LS003126), 0.1mg/mL DNase I, (Worthington Biochemical; Catalog#: LS002139), and 0.3mg/mL L-Cysteine (Sigma; Catalog#: C7352) and 10% 1M Hepes in HBSS, for 20 minutes at 37 °C. After digestion with Papain, the DRGs were washed twice with dissection media. Subsequently, they were incubated in a Collagenase solution for another 20 minutes at 37 °C, followed by two additional washes with dissection media. Next, the DRGs were gently triturated to create a single-cell suspension in complete Neurobasal A media (Thermo Fisher, Gibco; Catalog#: 12349015) supplemented with B-27^™^ Plus Supplement (Thermo Fisher, Gibco; Catalog#: A3582801) and GlutaMAX^™^ Supplement (Thermo Fisher, Gibco; Catalog#: 35050061). The cells were centrifuged at 300 g for 5 minutes, resuspended in complete Neurobasal A media, and plated on cover glasses coated with PDL-collagen (0.01mg/mL PDL (Sigma Catalog # P7405 and 0.2mg/mL Rat tail Collagen from Sigma Catalog # C7661). After being plated, the cells were incubated at 37 °C for 24 hours. For rFibulin-2 treatment, the cells were exposed to 2 μg/ml rFibulin-2 protein (R&D System Catalog # 9559-FB-050) or MQ water as a control at the time of seeding, and they were incubated for an additional 24 hours. After 24h incubation, cultures were used for electrophysiology recording or for IF experiments.

### Action potential recording and analysis

Whole-cell patch-clamp recordings in a current-clamp mode were performed using a MultiClamp 700B amplifier (Molecular Devices) from short-term cultures (24 h after plating) of isolated DRG neurons, visually identified with infrared video microscopy and differential interference contrast optics (Olympus BX51WI). Recordings were conducted at near-physiological temperature (33–34°C). In these conditions, the majority of cells analyzed were small/medium diameter IB4-positive neurons ^19,57^. The recording electrodes were filled with the following (in mM): 130 K-gluconate, 10 KCl, 0.1 EGTA, 2 MgCl_2_, 2 ATPNa_2_, 0.4 GTPNa, and 10 HEPES, pH 7.3. The extracellular solution contained (in mM): 145 NaCl, 3 KCl, 10 HEPES, 2.5 CaCl_2_, 1.2 MgCl_2_, and 7 glucose, pH 7.4. APs were evoked by a ramp-current injection (0.15 pA/ms) with a hyperpolarizing onset. AP threshold was defined as voltage where the AP rise speed reaches 5 mV/ms. The AP threshold was determined only from the first APs in the traces. AP rheobase was determined as current amplitude difference from resting membrane potential to threshold point. Rheobase charge transfer was the integration of the current over the time interval, which was from the point of ramp-current cross resting membrane potential to the first AP threshold point. AP amplitude was voltage difference between the threshold point and the AP peak. AP duration was defined as the time interval between AP rising and falling parts at the half-height level. The AP1-AP2 interval was defined as the time duration between the peaks of first and second APs. The fast afterhyperpolarization (fAHP) was the voltage difference between threshold point and the lowest point of AP hyperpolarization within 5 ms. All data were averaged over 5 trials for each cell. All chemicals for internal solution and bath solution were from Sigma-Aldrich. The Kv4 channel blocker Phrixotoxin-1 was from Alomone Labs.

### Determination of resting membrane potential, capacitance, and input resistance

Resting membrane potential (RMP) was measured immediately after whole-cell formation. Cell capacitance was determined by the amplifier’s auto whole-cell compensation function with slight manual adjustment to optimize the measurement if needed. The input resistance was estimated from the ramp-current evoked AP traces (Figure 3A): linear fitting 10-mV voltage interval with the corresponding ramp-current input in hyperpolarization (from 2 to 12 mV below RMP) or depolarization (from 5 to 15 mV above RMP) segments.

### Measurements of voltage-dependent K+ currents

Voltage-dependent K^+^ currents were recorded in voltage clamp mode using the same electrode solution as above. For step evoked K^+^ current measurement (Figure 4A-C), the bath solution was the same as above, except that equimolar choline chloride was used to replace the sodium chloride, and CdCl2 (100 μM) was added to block Ca2^+^ channels. Total K^+^ currents (I_Total_) were evoked by a series of test pulses (from −60 mV to + 50 mV with 500-ms duration and 10-mV step), preceded by a prepulse of −100 mV for 500 ms ^85^. I_K_ was isolated by changing the prepulse potential to −40 mV, and I_A_ = I_Total_ – I_K_. For ramp-evoked K^+^ measurement (Figure 4D-E), the internal solution and external solutions were the same as those of AP recordings, except that 1 μM TTX and 100 μM CdCl_2_ were added to block Na^+^ and Ca^2+^ channels, respectively. Voltage ramp from −100 to +20 mV (100 mV/s, and proceeded by a prepulse of −100 mV for 500 ms) was used to evoke K^+^ currents ^62^. The Kv4 currents (Phrixotoxin-1-sensitive component) were the difference between before and during Phrixotoxin-1 application. Series resistance compensation was enabled with 85–90% correction and 10–20 μs lag. Leak subtraction was done by P/5 protocol.

### Behavioral tests

For Von Frey testing, animals were habituated in boxes on an elevated metal mesh floor under stable room temperature and humidity. After acclimation for 1 hour, a set of von Frey filaments (Stoelting) with logarithmically increasing stiffness from 0.02 to 1.4 g were applied to the plantar surface of the hind-paw. Quick withdrawal or licking of the paw in response to the stimulus was considered a positive response. The 50% paw withdrawal threshold (PWT) was calculated using the up-down method. For hot plate tests, the mice were placed on a metal surface maintained at 50°C. The latency to lick or shake the hind paws were measured. The cutoff time was 40 seconds. For cold plate test, the mice were placed on a metal surface maintained at 0°C. The latency to lick or shake the hind paws were measured. The cutoff time was 20 seconds.

### Data availability

Raw and processed bulk RNA-seq data generated in this study have been deposited in the Gene Expression Omnibus (GEO) under accession number GSE317786. The single-cell RNA-seq atlas used for comparison is available under accession number GSE317728 ^39^.

## Supplementary Material

Supplement 1

Supplement 2

Supplement 3

Supplement 4

## Figures and Tables

**Key Resources:** 

Reagent type (species) or resource	Designation	Source or reference	Identifiers	Additional information
strain, strain background (Mus musculus)	C57BL/6	Envigo; Jackson Laboratory	Envigo: 027; JAX: 000664	Female and male, 2-3 months old
genetic reagent (M. musculus)	Fibulin-2 KO mice	Jackson Laboratory	JAX: 007914	B6.129S1-*Fbln2^tm1Chu^*/J
biological sample (M. musculus)	DRG tissue	This paper		L3-L5, L4-L5 DRGs isolated from adult
chemical compound, drug	Brefeldin-A	Invitrogen	00-4506-51	3μg/mL
chemical compound, drug	rhFibulin-2	R&D Systems	9559-FB	2μg/mL
antibody	Fibulin-2	Invitrogen	PA5-21640	WB (1:1000), IF(1:250)
antibody	VAMP5	Synaptic system	176-011	IF (1:100)
antibody	CD9	Invitrogen	MA3-005	WB (1:500)
antibody	VAMP1/2/3	Synaptic system	104-104	IF (1:100)
antibody	GAPDH	Cell Signaling	5174s	WB (1:5000)
antibody	FABP7	Invitrogen	PA5-24949	IF (1:1000)
antibody	GM130	BD	610822	WB (1:1000), IF: (1:200)
antibody	NEUN	SySY	266-011	IF (1:1000)
antibody	TUJ1	Biolegend	801202	IHC (1:1000)
Antibody	TH	Sigma	AB1542	1:1000
Antibody	TRPV1	Allomone lab	ACC-030-GP	1:500
antibody	Chicken-Anti-NF200	Abcam		1:1000
Probe	IB4	Vectror-Shield	FL-1201-.5	(1:200)
antibody	Kv4.2 (K57/1)	DSHB	K57/1	(1:500)
antibody	Kv4.3	Alomone Lab	APC-017	(1:1000)
antibody	Rabbit anti-PGP9.5 (polyclonal)	LS Bio	LS-B5981-50	IHC (1:500)
antibody	Secondary antibodies Alexa Fluor 488/594/647	Invitrogen	Various	IHC (1:500)
commercial assay, kit	RNeasy Micro Kit	QIAGEN	74004	RNA extraction
commercial assay, kit	High-Capacity cDNA Reverse Transcription Kit	Thermo Fisher	4368814	cDNA synthesis
Commercial kit	BCA	Thermo Fisher, Pierce	23227	For Protein qunatification
Commercial Kit	Total exosome Isolation Kit	Thermo Fisher	4478359	For EV siolation
Commercial Kit	Thermo Signal Dura	Thermo Fisher	34076	ECL
commercial assay, kit	PowerUp^™^ SYBR^™^ Green Master Mix	Applied Biosciences	4309155	qPCR
chemical compound	DAPI	Sigma-Aldrich	D9542	(300 nM) nuclear stain
commercial assay, kit	ProLong^™^ Gold Antifade Mountant	Invitrogen	P36930	For mounting fluorescent samples
Chemical compound	Ponceau	Thermo Fisher	A40000279	For western blot
chemical compound	PFA (paraformaldehyde)	Various		4% used for fixation
chemical compound	OCT compound	Tissue-Tek		For cryosectioning
chemical compound	HBSS	Thermo Fisher, Gibco	14175-079	Dissection medium
chemical compound	HEPES	Thermo Fisher, Gibco	15630080	Buffering agent
chemical compound	Papain	Worthington Biochemical	LS003126	For tissue dissociation
chemical compound	L-cysteine	Sigma	C7352	Added to dissociation mix
chemical compound	DNase I	Worthington Biochemical	LS002139	For DNA degradation during dissociation
chemical compound	Collagenase	Sigma	C6885	Enzyme for tissue dissociation
chemical compound	Neurobasal^™^-A Medium	Thermo Fisher, Gibco	12349015	Culture medium
chemical Compound	DMEM	Gibco	11054020	SGC Culture Medium
commercial assay, kit	B-27^™^ Plus Supplement	Thermo Fisher, Gibco	A3582801	Culture supplement
commercial assay, kit	GlutaMAX^™^ Supplement	Thermo Fisher, Gibco	35050061	Glutamine substitute
chemical compound	Poly-D-lysine	Sigma	P7405	Coating coverslips
Chemical compound	Rat-Tail collagen	Sigma	C7661	Coating coverslip
software, algorithm	Fiji	Schindelin et al., 2012	RRID:SCR_002285	Image analysis
software, algorithm	QuantStudio 6 Flex System	Thermo Fisher		For qPCR analysis
software, algorithm	Imaris	Oxford Instruments	v9.7	For High Resolution Imaging
equipment	Nikon-NSPARC	Nikon		Confocal microscope
equipment	ECLIPSE Ti2 microscope	Nikon		Imaging cultured neurons
equipment	Jeol JEM-1400 Plus	Jeol		Transmission Electron Microscopy
				

## Highlights:

- Fibulin-2 is secreted by cultured SGCs
- Fibulin-2 reduces sensory neuron excitability and firing frequency
- Fibulin-2 acts by modulating Kv4-mediated potassium currents
- Loss of Fibulin-2 heightens mechanical, heat and cold sensitivity in mice
