# Supplementary material for: Satellite Glial Cells Control Sensory Neuron Excitability via the Release of Fibulin-2
Source: bioRxiv. 2026 Feb 15:2026.02.13.705760. Preprint. [Version 1] doi: 10.64898/2026.02.13.705760 (PMC12918954; doi:10.64898/2026.02.13.705760)
Supplement: Supplement 4 [file NIHPP2026.02.13.705760v1-supplement-4.pdf]

bioRxiv preprint doi: https://doi.org/10.64898/2026.02.13.705760; this version posted February 16, 2026. The copyright holder for this preprint (which was not certified by peer review) is the author/funder, who has granted bioRxiv a license to display the preprint in perpetuity. It is made available under aCC-BY 4.0 International license.

Supplementary Figure 1

A. Representative images of SGC cultures stained for the SGCs markers FABP7, and GS, the Schwann cell marker S100B, the neuron marker TUJ1 and the fibroblast marker PDGFRα. Scale bar 100μm.

B. RT-qPCR analysis of SGC cultures for known SGCs markers (*Fabp7, Glul, GjaI, Ednrb, Gfap*), Schwann cells (*Scn7a, Mpz, Ncam*), macrophages (*Aif, Itgam, Cx3cr1*), neurons (*Nfeh, Prph*), and fibroblasts (*Fgf13, Fgf9*). N=2.

C. Transcriptional similarity between cultured cells and primary tissue cell types. Heatmap showing Spearman correlation coefficients between bulk RNA-seq profiles from cultured samples (columns) and pseudobulk expression profiles from major cell classes in the single-cell atlas (rows). Correlation was computed using 5,000 highly variable genes following joint TMM normalization. Values within cells indicate correlation coefficients; color scale ranges from blue (low correlation) to red (high correlation). Hierarchical clustering was performed using Euclidean distance.

D. Bar plot showing mRNA expression levels (counts per million, CPM) for a panel of cell-type-specific marker genes in bulk RNA-seq from primary SGC cultures. Bars are colored by cell type: Glial/SGC (blue), Schwann (green), Neuronal (black), Fibroblast (purple), Endothelial (orange), Mural (red), and Immune (yellow). Individual sample values are shown as points; circles indicate serum-free (24h) samples, triangles indicate serum-containing samples. Error bars represent standard deviation.

E. Representative western blot (from 3 independent experiments) of DRG and SGC lysate from WT and Fibulin-2 KO mice, probed for Fibulin-2. GAPDH and Ponceau are used as loading controls.

bioRxiv preprint doi: https://doi.org/10.64898/2026.02.13.705760; this version posted February 16, 2026. The copyright holder for this preprint (which was not certified by peer review) is the author/funder, who has granted bioRxiv a license to display the preprint in perpetuity. It is made available under aCC-BY 4.0 International license.

F. Representative western blot of SGC-CM from WT and Fibulin-2 KO mice, probed for Fibulin-2. Ponceau staining is used as a loading control.

G. Ponceau staining of western blot showed in Fig. 1I for protein loading control.

Supplementary Figure 2

Expression of potassium and TRP channels across neuronal subtypes using single cell RNAseq atlas of the mouse DRG [39]. Dot plot showing expression of voltage-gated potassium channel subunits (*Kcnd1*, *Kcnd2*, *Kcnd3*, *Kcna4*), TRP channels (*Trpv1*, *Trpm8*), and neuronal markers (*Calca*, *Nefh*) across identified neuronal clusters. Dot size represents the percentage of cells expressing each gene; color intensity indicates average expression level.

Supplemental Data File 1

List of proteins identified in SGC-CM using mass spectrometry. Three blank media (blank 1, blank 2, and blank 3) were used as controls. SGCs-CM samples were collected from three independent biological experiments (SGC_CM1, SGC_CM2, and SGC_CM3). Columns include Proteins identifier (Identified proteins, Accession numbers, Alternate ID, molecular weights), Total spectrum count quantified using mass spectrometry.

Supplemental Data File 2

List of proteins identified in the SGC cell pellet using mass spectrometry. Columns include Proteins identifier (Protein Name, Accession numbers, Alternate ID, molecular weight), Total spectrum count was quantified using mass spectrometry.

Supplemental Data File 3

Proteins identified in the SGCs-CM were filtered using stringent criteria: t-test p-value < 0.05, fold change (SGC-CM/Blank) > 2, maximum blank signal ≤ 5, and mean SGC-CM signal ≥ 10. Expression data from single-cell RNA sequencing of mouse DRG were integrated for genes present in the dataset. Columns include protein identifiers (ProteinName, AccessionNumber, GeneSymbol), mass spectrometry quantification (blank_1–3, SGC_CM_1–3, with corresponding means), statistical metrics (T_Test_pvalue, FC_NF_vs_Blank), and single-cell expression values (SGC_expr, Max_other_expr, Max_other_celltype, SGC_ratio, and per-cell-type expression). SGC_ratio represents SGC expression divided by the maximum expression in other cell types (with 0.1 pseudocount).

Supplementary Figure 1

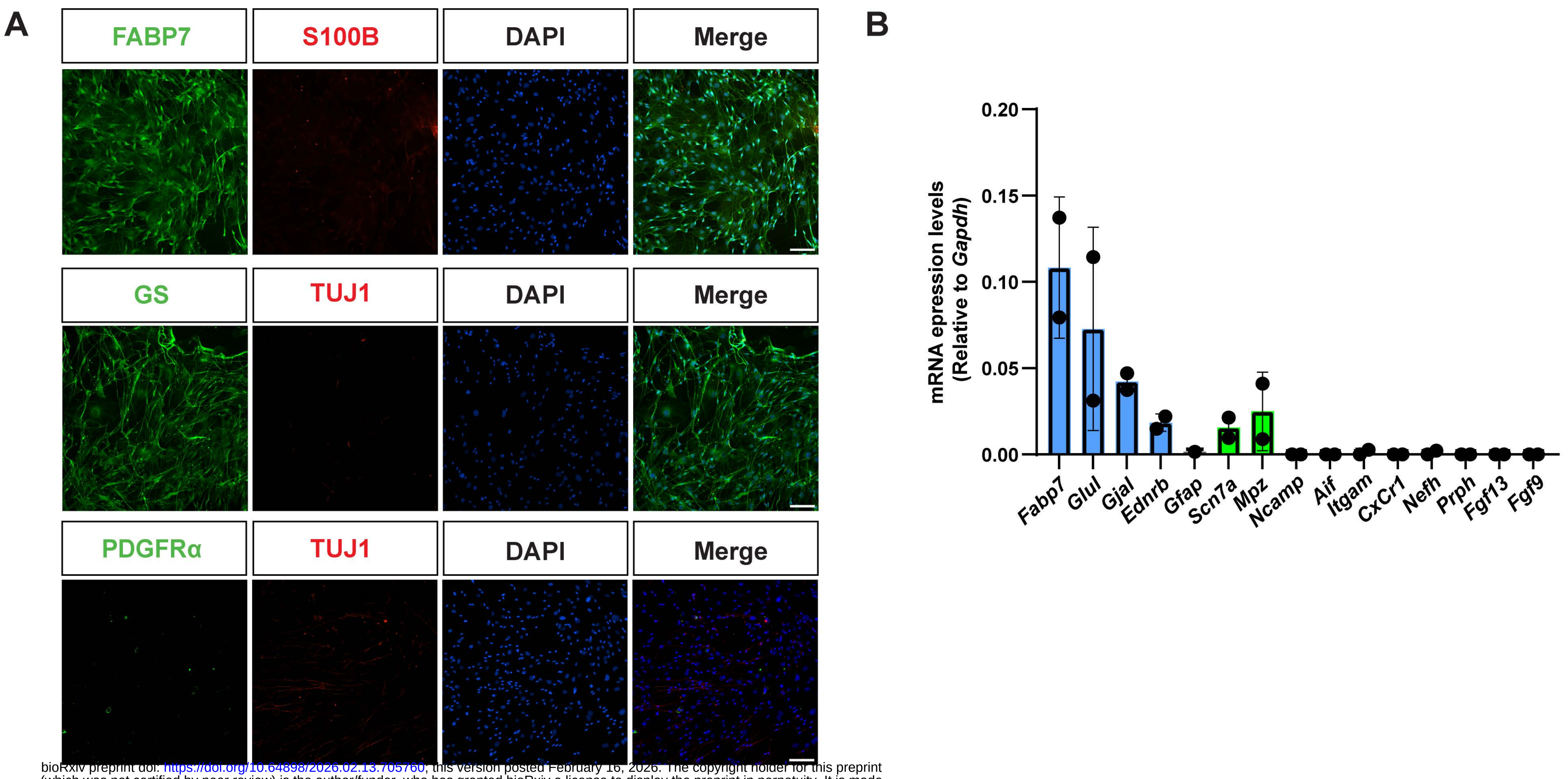

bioRxiv preprint doi: https://doi.org/10.64898/2026.02.13.705760; this version posted February 16, 2026. The copyright holder for this preprint (which was not certified by peer review) is the author/funder, who has granted bioRxiv a license to display the preprint in perpetuity. It is made available under aCC-BY 4.0 International license.

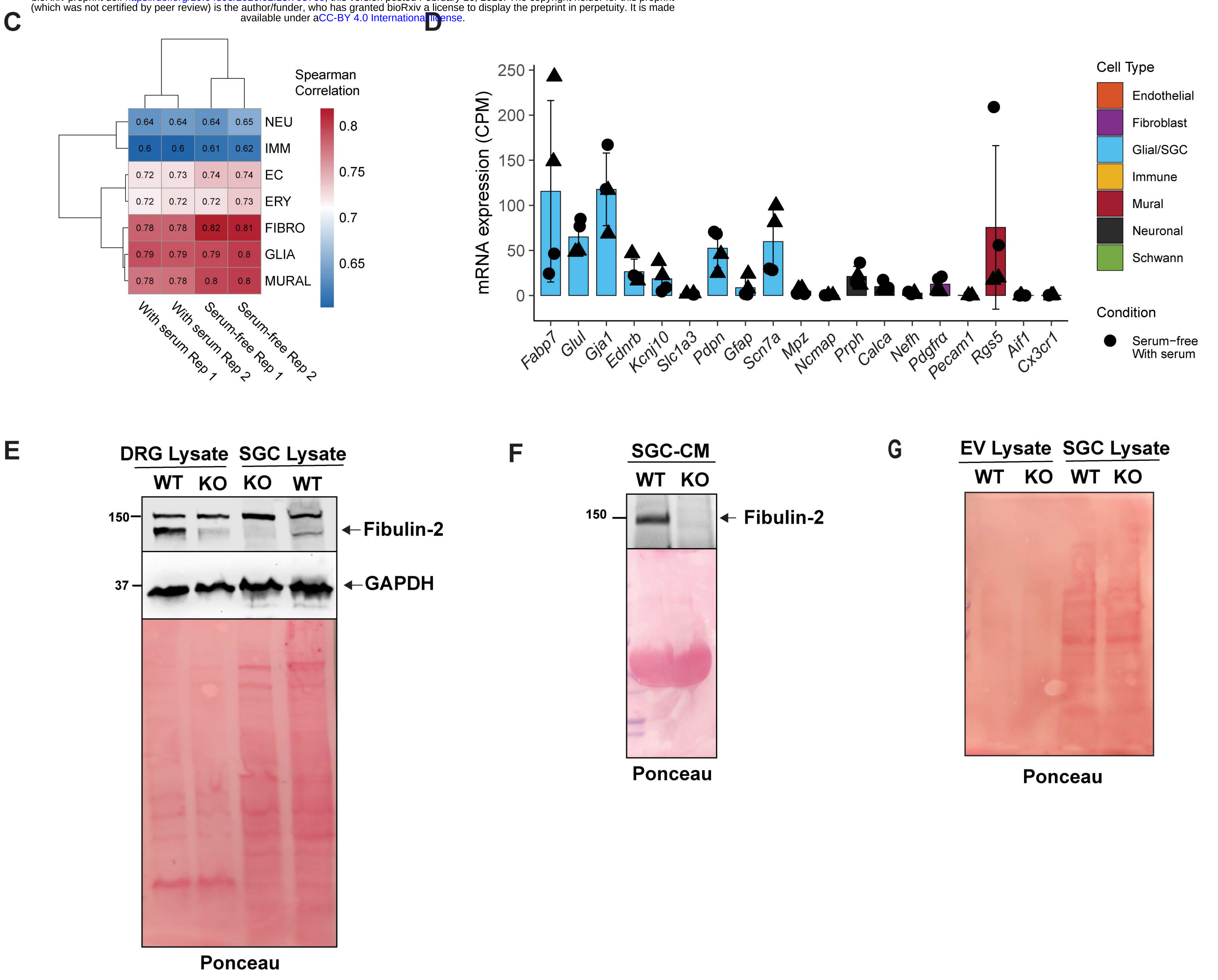

Supplementary Figure 2

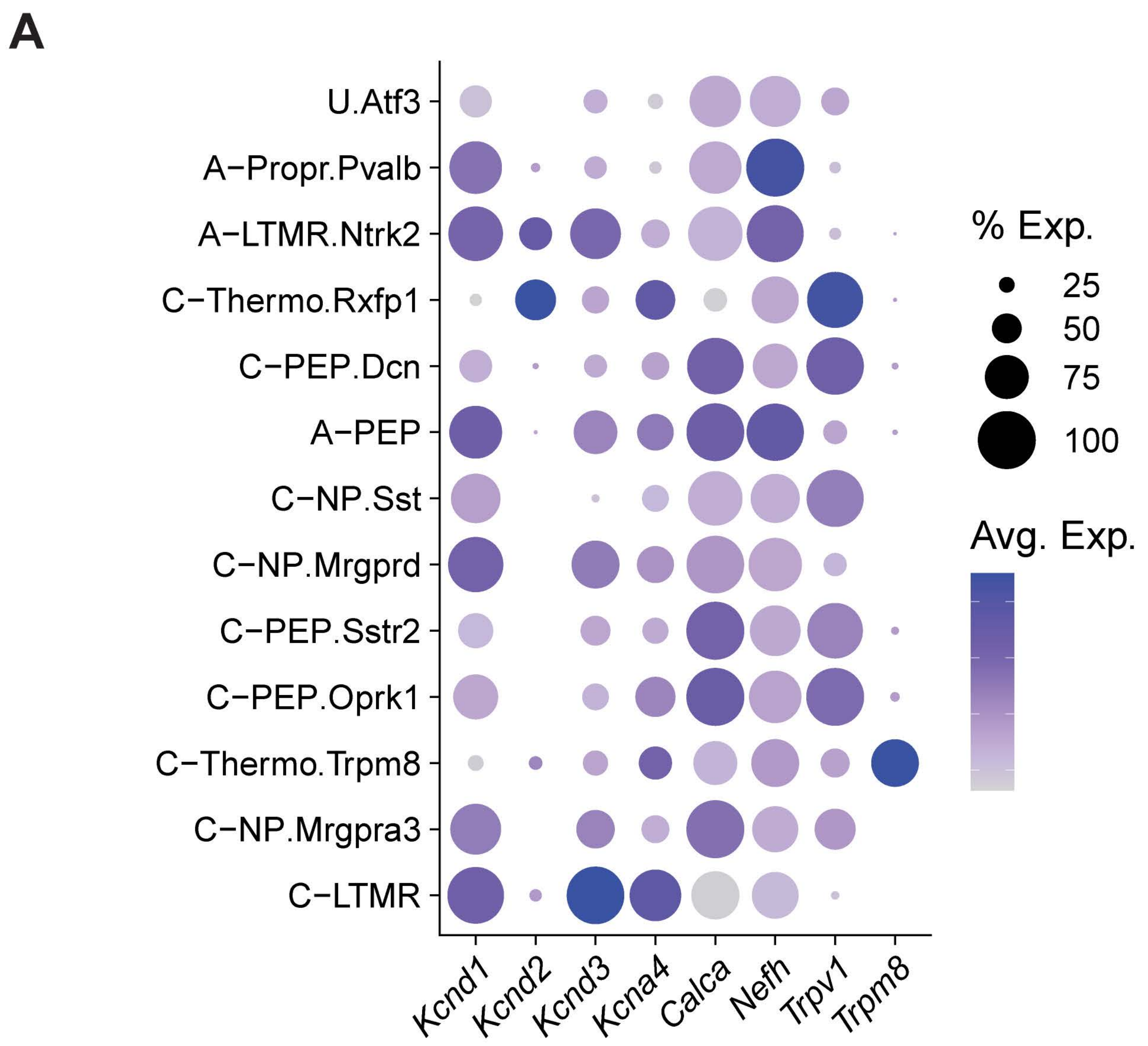

bioRxiv preprint doi: https://doi.org/10.64898/2026.02.13.705760; this version posted February 16, 2026. The copyright holder for this preprint (which was not certified by peer review) is the author/funder, who has granted bioRxiv a license to display the preprint in perpetuity. It is made available under aCC-BY 4.0 International license.
